# Impact of timing of primary ileocecal resection on prognosis in patients with Crohn’s disease

**DOI:** 10.1093/bjsopen/zrad097

**Published:** 2023-09-29

**Authors:** Evelien M J Beelen, Jeanine H C Arkenbosch, Nicole S Erler, Jasmijn A M Sleutjes, Frank Hoentjen, Alexander G L Bodelier, Gerard Dijkstra, Marielle Romberg-Camps, Nanne K de Boer, Laurents P S Stassen, Andrea E van der Meulen, Rachel West, Oddeke van Ruler, C Janneke van der Woude, Annemarie C de Vries

**Affiliations:** Department of Gastroenterology and Hepatology, Erasmus University Medical Center, Rotterdam, The Netherlands; Department of Gastroenterology and Hepatology, Erasmus University Medical Center, Rotterdam, The Netherlands; Department of Biostatistics, Erasmus University Medical Center, Rotterdam, the Netherlands; Department of Gastroenterology and Hepatology, Erasmus University Medical Center, Rotterdam, The Netherlands; Department of Gastroenterology and Hepatology, Radboud University Medical Center, Nijmegen, The Netherlands; Division of Gastroenterology, Department of Medicine, University of Alberta, Edmonton, Alberta, Canada; Department of Gastroenterology and Hepatology, Amphia Hospital, Breda, The Netherlands; Department of Gastroenterology and Hepatology, University Medical Center Groningen, Groningen, The Netherlands; Department of Gastroenterology and Hepatology, Zuyderland Medical Center, Sittard-Geleen, The Netherlands; Department of Gastroenterology and Hepatology, AGEM Research Institute, Amsterdam University Medical Center, Vrije Universiteit Amsterdam, Amsterdam, The Netherlands; Department of Surgery, Maastricht University Medical Center, Maastricht, The Netherlands; Department of Gastroenterology and Hepatology, Leiden University Medical Center, Leiden, The Netherlands; Department of Gastroenterology and Hepatology, Fransiscus Gasthuis & Vlietland, Rotterdam, The Netherlands; Department of Surgery, IJsselland Hospital, Capelle aan den IJssel, The Netherlands; Department of Gastroenterology and Hepatology, Erasmus University Medical Center, Rotterdam, The Netherlands; Department of Gastroenterology and Hepatology, Erasmus University Medical Center, Rotterdam, The Netherlands

## Abstract

**Background:**

The advantage of early ileocecal resection after Crohn’s disease diagnosis is a matter of debate. This study aims to assess the timing of ileocecal resection on prognosis, after correction for possible confounders.

**Methods:**

Patients with Crohn's disease with primary ileocecal resection between 2000 and 2019 were included in a retrospective multicentre cohort. The primary endpoint was endoscopic recurrence (Rutgeerts score ≥i2b) within 18 months. Secondary endpoints were escalation of inflammatory bowel disease medication within 18 months and re-resection during follow-up. The association between timing of ileocecal resection and these endpoints was investigated using multivariable proportional hazard models, corrected for covariates including Montreal classification, postoperative prophylaxis, smoking, indication for surgery, medication before ileocecal resection, perianal fistulas, surgical approach, histology, length of resected segment and calendar year.

**Results:**

In 822 patients ileocecal resection was performed after a median of 3.1 years (i.q.r. 0.7–8.0) after Crohn's disease diagnosis. The lowest incidence of endoscopic recurrence, escalation of inflammatory bowel disease medication and re-resection was observed for patients undergoing ileocecal resection shortly after diagnosis (0–1 months). After correction for covariates, patients with ileocecal resection at 0, 4 and 12 months after diagnosis had a cumulative incidence of 35 per cent, 48 per cent and 39 per cent for endoscopic recurrence, 20 per cent, 29 per cent and 28 per cent for escalation of inflammatory bowel disease medication and 20 per cent, 30 per cent and 34 per cent for re-resection, respectively. In the multivariable model ileocolonic disease (HR 1.39 (95 per cent c.i. 1.05 to 1.86)), microscopic inflammation of proximal and distal resection margins (HR 2.20 (95 per cent c.i. 1.21 to 3.87)) and postoperative prophylactic biological and immunomodulator (HR 0.16 (95 per cent c.i. 0.05 to 0.43)) were associated with endoscopic recurrence.

**Conclusion:**

The timing of ileocecal resection was not associated with a change of disease course; in the multivariable model, the postoperative recurrence was not affected by timing of ileocecal resection.

## Teaser

This study aims to assess the impact of timing of ileocecal resection (ICR) on postoperative prognosis of Crohn’s disease (CD) in a multivariable model. After correction for possible confounders, patients with early and late ICR have an equally beneficial short- and long-term postoperative prognosis. Therefore, timing of ICR in CD patients is not associated with a change of disease course.

## Introduction

Despite the availability and increased use of immunomodulating therapy, intestinal resection remains an important treatment modality for Crohn’s disease (CD)^1,2^. Ileocecal resection (ICR) is the most common intestinal resection in CD^3^. ICR can provide immediate relief of symptoms in CD patients with ileal or ileocecal disease and may induce long-term remission. Data from the pre-biologic era show 80 per cent of patients are still symptom-free after 5 years, and 48 per cent of patients 10 years after ICR^4^. However, early endoscopic postoperative recurrence rates, preceding clinical symptoms, are estimated at 60–85 per cent and up to 35 per cent of CD patients require re-resection during long-term follow-up^2,5–7^. Identification of CD patients at high risk of postoperative recurrence remains a challenge^8^. Active smoking was found to be an individual and consistent risk factor in several studies^9^. Other risk factors proposed by current literature are penetrating disease and prior intestinal surgery^8,10^. Nonetheless, available studies do not show consistent results^8,10–13^ and large studies investigating risk factors in multivariable models are necessary.

The introduction and widespread availability of immunomodulating therapy might have led to postponement and reservation of surgical interventions for patients with refractory disease. However, the landmark randomized controlled ‘LIR!C’ trial has shown that laparoscopic ICR may be considered a valid alternative to step-up treatment with infliximab in patients with non-complicated (non-stricturing, non-penetrating) ileal disease activity, with regard to the endpoints of patient-reported quality of life and cost-effectiveness at 12 months^14,15^. Nevertheless, the advantage of early ICR after CD diagnosis, as compared to ICR during a later stage of disease, remains a matter of debate. Early ICR performed shortly after CD diagnosis would fit the idea that this period hosts the largest window of opportunity to change the disease course of CD. According to this hypothesis, early ICR could prolong clinical remission, reduce the need for long-term exposure to immunomodulators and biologicals, and reduce the risk of complications caused by progressive bowel damage due to chronic intestinal inflammation^16,17^. Data comparing clinical outcome after ICR at different periods of disease duration are scarce and limited to a few cohorts^18–20^. Furthermore, the definition of early ICR is arbitrary and the study populations are small, heterogeneous and mostly not corrected for confounding factors (including disease severity, smoking, prophylactic medication), hampering interpretation of the results.

Therefore, this study aimed to assess the impact of timing of ICR on postoperative prognosis of CD adjusted for confounding factors.

## Materials and methods

### Study design and study population

A retrospective, multicentre study was performed in patients who had undergone an ICR for the indication of CD between January 2000 and December 2019. Eligible patients were identified from local hospital pathology databases of the participating centres, including six academic and four teaching hospitals. All CD patients aged ≥16 years with ileal disease localization with or without colon involvement and who underwent a primary ICR with one- or two-stage ileocolonic anastomosis were included. In case of a two-stage procedure (with ileocolonic anastomosis after temporary ileostomy) the moment of anastomosis was chosen as baseline. Exclusion criteria were a permanent stoma, clinical or endoscopic recurrence before intestinal continuity was restored, prior intestinal resections, other indications for ICR (for example, malignancy), and absence of follow-up data. This study was performed in accordance with the declaration of Helsinki and its protocol was assessed and approved by the Medical Ethical Research Committee of the Erasmus University Medical Centre on 10 November 2017.

### Data collection

Electronic patient files were searched to include demographics, patient-related factors (for example, smoking habit and family history of inflammatory bowel disease (IBD)), disease-related factors (for example, medication use and Montreal classification) and surgery-related factors (for example, indication for ICR, surgical technique, length of the resected segment and histologic inflammation of resection margins). The initiation or continuation of IBD medication after surgery for the prevention of postoperative recurrence was also noted. Data were collected until loss to follow-up, re-resection, death or end of study.

### Endpoints

The primary endpoint was endoscopic recurrence (defined as Rutgeerts score ≥ i2b, ≥ 5 aphthous ulcers with normal intervening mucosa or skip areas of larger lesions in the neo-terminal ileum^21^) and/or radiologic recurrence (CD disease activity as assessed by a local radiologist on ultrasonography, CT or MRI scan) within 18 months after ICR. The secondary endpoints were escalation of IBD medication within 18 months after ICR (defined as the need for initiation of or switch to mesalazine, corticosteroids, immunomodulators or biologicals for symptomatic disease after ICR) and re-resection during long-term follow-up.

### Statistical analysis

Categorical variables were described using frequencies and percentages and continuous variables were described using median and 1st and 3rd quartiles (Q1–Q3) and range for non-normally distributed variables. All calculations were performed in R version 4.0.3 (R Core Team 2020) and with the help of the package JointAI^22^.

Risk factors for endoscopic recurrence were analysed using a multivariable proportional hazard model. The included covariates were timing of ICR, smoking status, age at diagnosis, disease localization (Montreal L), disease behaviour (Montreal B), prophylactic postoperative medication use, indication for surgery (non-complicated disease refractory to medical therapy or ICR instead of therapy escalation, stricturing disease, penetrating disease or another indication), gender, perianal fistulas, immunomodulator use prior to ICR, biological use prior to ICR, surgical approach (laparoscopy or laparotomy), histologic inflammation of the surgical resection margins (proximal (ileal) inflammation, distal (colonic) inflammation or both), length of the resected segment and the calendar year of ICR. A limited number of these variables were selected to display differences in cumulative incidence of endoscopic and/or radiologic recurrence. These variables were selected based upon current literature and/or statistical significance in the model.

The association between timing of ICR and the postoperative endpoints was investigated using multivariable proportional hazards models, adjusted for all above-mentioned covariates, in the multivariable model. To avoid bias by creating predefined categories, timing of ICR was analysed as a continuous variable. The characteristics of patient groups with different timings of ICR were subsequently assessed in detail. These groups were defined according to the results of the primary analysis. Due to the lower number of events for re-resection, the model for this outcome only included six covariates (timing of ICR, active smoking, age at diagnosis, Montreal L, Montreal B, postoperative prophylactic medication). To allow for a non-linear effect of the time between diagnosis and surgery we used a natural cubic spline with six degrees of freedom. To be able to include patients in the analysis for whom some covariates were missing, the models were fitted in the Bayesian framework, which allowed us to simultaneously fit the three models and impute missing covariates^23^. In addition, the models were refitted with timing of ICR as the only explanatory variable.

A sensitivity analysis for the endpoint of severe endoscopic recurrence, defined as Rutgeerts score ≥ i3 (diffuse aphthous ileitis with diffusely inflamed mucosa^6^), was also performed. Due to the lower number of events in this sensitivity analysis, the multivariable model included six covariates (timing of ICR, active smoking, age at diagnosis, Montreal L, Montreal B, postoperative prophylactic medication).

## Results

### Study population

Overall, 835 patients with CD who underwent a primary ICR between 2000 and 2019 and were followed afterwards in the hospitals were included. All pathology reports were captured in the nationwide Dutch PALGA database (see website as reference, https://www.palga.nl). This ascertains full coverage of these cases.

Four patients were excluded because they had a permanent stoma and nine patients were excluded because they had recurrence before the stoma was removed. The total cohort of patients analysed comprised 822 patients (317 (38.6 per cent) male, median age 32.3 years (Q1–Q3 24.1–45.1, range 16.0–78.0)) who had undergone primary ICR (*Table 1*). Patient inclusion over time is included in *Fig. S1*. One-stage ileocolonic anastomosis was performed in 781 (95.0 per cent) patients and a two-stage procedure, with ileocolonic anastomosis after temporary ileostomy, was performed in 41 (5.0 per cent) patients. The affected disease location at the timing of surgery was limited to the ileum in 523 (63.6 per cent) patients, whereas 299 (36.4 per cent) patients had ileal and caecal or ascending colon involvement. In 224 (27.3 per cent) patients the indication for ICR was non-complicated disease, refractory to current treatment or ICR as alternative to therapy escalation. In 398 (48.4 per cent) patients the indication was a symptomatic CD stricture, and in 185 (22.5 per cent) patients penetrating disease causing an intra-abdominal fistula or abscess. In a small proportion of patients, 15 (1.8 per cent), another indication for ICR was recorded, 5 (0.6 per cent) patients with suspected appendicitis and 10 (1.2 per cent) diagnostic laparoscopy or laparotomy. In total, 285 (34.7 per cent) patients were active smokers at the time of ICR. At ICR, 281 (34.2 per cent) patients were immunomodulator naïve, 456 (55.4 per cent) patients biological naïve, and 240 (29.2 per cent) patients were naïve to both.

**Table 1:** Characteristics of the study population. Shown are median (i.q.r.) or count (proportion)

	Total population (*N* = 822)	Missing
Sex, male	317 (38.6%)	0
Age at ICR in years, median	32.3 (24.1–45.1)	0
Positive family history of IBD	167 (20.3%)	275 (33.5%)
Active smoking	285 (34.7%)	53 (6.4%)
BMI, median	22.6 (19.8–25.7)	239 (29.1%)
Age at diagnosis in years	25.5 (19.9–37.0)	0
**Montreal L**		
Ileum	523 (63.6%)	0
Ileocolonic	299 (36.4%)	
**Montreal B**		
Non-stricturing, non-penetrating	177 (21.5%)	0
Stricturing	400 (48.7%)	
Penetrating	245 (29.8%)	
Perianal fistulas	97 (11.8%)	0
Biological use prior to ICR	366 (44.5%)	0
Immunomodulator use prior to ICR	541 (65.8%)	0
**Indication for ICR**		
Non-complicated disease refractory to therapy/instead of step-up	224 (27.3%)	0
Stricture	398 (48.4%)	
Fistula/abscess	185 (22.5%)	
Other*	15 (1.8%)	
**Surgical approach**		
Laparotomy	362 (44.0%)	34 (4.1%)
Laparoscopy	426 (51.8%)	
**Microscopic inflammation of resection margin**		
No inflammation	386 (47.0%)	223 (27.1%)
Proximal inflammation	135 (16.4%)	
Distal inflammation	37 (4.5%)	
Both sides inflammation	41 (5.0%)	
Length of resected segment in cm, median	25.0 (18.0–33.5)	63 (7.7%)
**Type anastomosis**		
Side-to-side	597 (72.6%)	100 (12.2%)
End-to-side	51 (6.2%)	
End-to-end	74 (9.0%)	
**Prophylactic postoperative medication**		
None/5ASA/corticosteroid	514 (62.5%)	2 (0.2%)
Immunomodulator	187 (22.8%)	
Biological	69 (8.4%)	
Immunomodulator and biological	50 (6.1%)	

* Other indications for ileocecal resection (ICR) comprised: suspected appendicitis in five (0.6%) patients, diagnostic laparoscopy or laparotomy in ten (1.2%) patients. IBD, inflammatory bowel disease.

### Postoperative disease recurrence and medical escalation

ICR was performed at a median of 3.1 years (Q1–Q3 0.7–8.0, range 0–46) after CD diagnosis. Median total follow-up was 5.6 years (Q1–Q3 2.5–10.7, range 0.1–19.9). Postoperative endoscopy or radiologic imaging within 18 months after ICR was performed in 527 patients, in whom endoscopic and/or radiologic recurrence was diagnosed in 218 (41.4 per cent) patients, after a median of 6.5 months (Q1–Q3 4.1–10.1, range 0.1–18.0). In 256 (31.1 per cent) CD patients, IBD medication was escalated after a median of 7.6 months (Q1–Q3 5.1–11.3, range 0.2–203.2). During total follow-up, 136 patients (26.5 per cent) underwent a re-resection (25 (18.5 per cent) ileum, 12 (8.9 per cent) colon, 98 (72.6 per cent) ileocolonic) after a median of 4.4 years (Q1–Q3 1.8–7.7, range 0.1–16.3).

### The impact of timing of ileocolic resection

The lowest incidence of endoscopic recurrence, escalation of IBD medication within 18 months after ICR and re-resection during total follow-up, was observed for patients undergoing ICR immediately at or shortly after diagnosis (0–1 months). After 1 month, recurrence rates increased and stabilized when the interval between initial diagnosis and ICR exceeded 4 months. For ICR during the rest of the disease course, no evidence for a difference in postoperative prognosis was found. Figures corresponding to the uncorrected model can be found in *Fig. S2*.

**Fig. 1:**
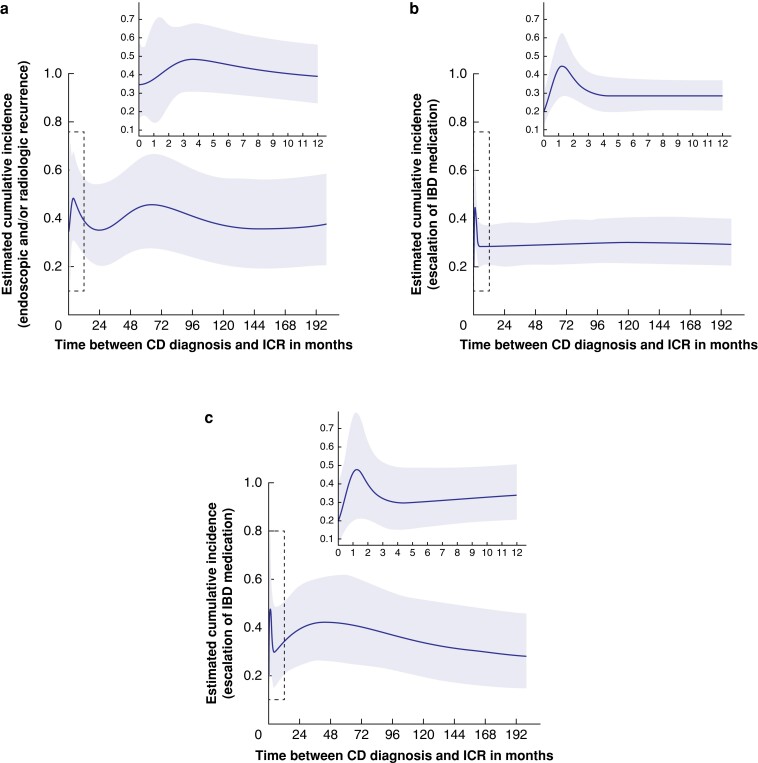
**Visualization of the effect of the timing of ileocecal resection (ICR) on recurrence, modelled with natural cubic splines**. Shown is the expected cumulative incidence 18 months after ileocecal resection (ICR) for endoscopic and/or radiologic recurrence (*n* = 527) and escalation of inflammatory bowel disease (IBD) medication (*n* = 822) (panels **a** and **b**), and after total follow-up (17 years) for surgical recurrence (*n* = 822) (panel **c**), adjusted for other covariates. The shaded area represents the corresponding 95% c.i. The inset plots zoom in on the first 12 months. CD, Crohn's disease.

After correction for possible confounding factors in a multivariable model documented in *Table 2*, similar trends were observed, although differences in recurrence rates between patients with ICR shortly after diagnosis (0–1 months) *versus* those performed at later stages of the disease course were smaller (*Fig. 1*). The cumulative incidence of endoscopic and/or radiologic recurrence at 18 months was estimated at 34.6 per cent, 48.2 per cent and 39.0 per cent for patients who underwent ICR, respectively, at 0, 4 and 12 months after diagnosis (*Fig. 1*). The estimated incidence of escalation of IBD medication at 18 months, for ICR performed 0, 4 and 12 months after diagnosis, was 20.2 per cent, 28.8 per cent and 28.3 per cent, respectively. Incidence of re-resection during total follow-up for patients who underwent ICR at 0, 4 and 12 months was 19.8 per cent, 29.8 per cent and 34.0 per cent, respectively (*Fig. 1*). To evaluate clinical characteristics of patients undergoing ICR during different stages of disease, the population was subdivided into three groups based on these curves. In the first group patients with ICR between 0 and 1 months after diagnosis were grouped (78 patients), then patients with ICR 1–4 months after diagnosis (52 patients) and thirdly patients with ICR >4 months after diagnosis (692 patients). Clinical and surgical characteristics of the different subgroups are displayed in *Table S1*.

**Table 2:** Results from the multivariable proportional hazards models for endoscopic and/or radiologic recurrence (*n* = 527). Shown are HRs and corresponding 95% c.i.

	Endoscopic and/or radiologic recurrence	
	HR	95% c.i.
Timing of ICR	*	
Active smoking	1.21	0.90–1.62
Age at diagnosis	1.00	0.99–1.01
**Montreal L**		
Ileum	Ref	
Ileocolonic	**1.39**	**1.05–1.86**
**Montreal B**		
Non-stricturing, non-penetrating	Ref	
Stricturing	0.82	0.48–1.39
Penetrating	0.77	0.42–1.35
**Postoperative prophylactic medication**		
None/5ASA/corticosteroid	Ref	
Immunomodulator	**0.60**	**0.40–0.87**
Biological	0.70	0.42–1.17
Immunomodulator and biological	**0.16**	**0.05–0.43**
**Indication for surgery**		
Non-complicated, refractory disease/therapy escalation	Ref	
Stricturing disease	1.03	0.62–1.67
Penetrating disease	0.91	0.50–1.75
Other	**0.13**	**0.01–0.93**
Gender, female	0.84	0.64–1.12
Perianal fistula	1.21	0.80–1.83
Immunomodulator use prior to surgery	1.24	0.84–1.83
Biological use prior to surgery	1.03	0.73–1.47
Surgical approach, laparotomy	0.98	0.70–1.37
**Microscopic activity at resection margin**		
Free of inflammation	Ref	
Proximal inflammation	1.39	0.94–2.05
Distal inflammation	1.32	0.67–2.43
Both sides inflammation	**2.20**	**1.21–3.87**
Length of resected segment in cm	1.00	0.99–1.01
Year of ICR	1.03	0.99–1.07

* HR of the non-linear effect component has no meaningful interpretation. Statistically significant results are shown in bold. ICR, ileocecal resection.

In the sensitivity analysis of severe endoscopic recurrence, using Rutgeerts score ≥ i3 as cut-off for the outcome endoscopic and/or radiologic recurrence, comparable results were found regarding the effect of timing of ICR on recurrence rates. The estimated incidence of severe endoscopic recurrence was 23 per cent, 38 per cent and 31 per cent for ICR at 0, 4 and 12 months after CD diagnosis, respectively (*Fig. S3*).

### Risk factors for endoscopic recurrence

**Fig. 2:**
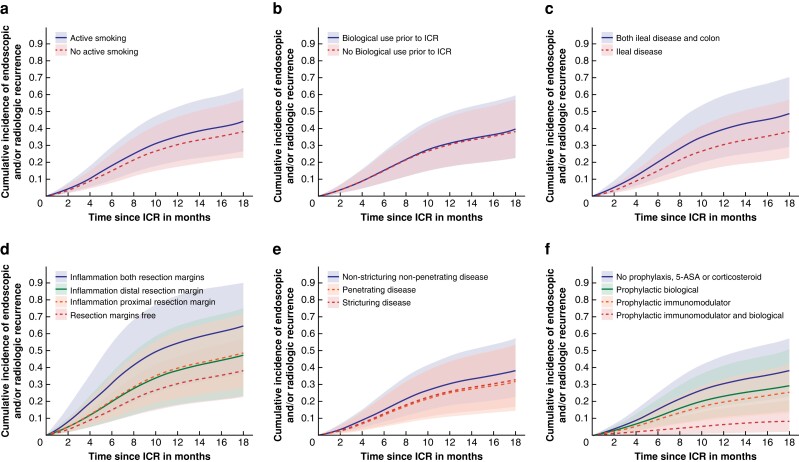
**The expected cumulative incidence of endoscopic and/or radiologic recurrence after ileocecal resection (ICR), displayed for a selection of covariates (all other covariates set to reference values)**. Visualized are the covariates smoking status **a**, biological use prior to ICR **b**, disease localization **c**, microscopic inflammation of the resection margins **d**, disease behaviour **e**, and postoperative prophylactic medication **f**. The shaded areas represent the corresponding 95% c.i.

Results of the multivariable proportional hazards model, with HRs and 95 per cent c.i., for endoscopic and/or radiologic recurrence are displayed in *Table 2*. In the multivariable model, ileocolonic disease (HR 1.4(95 per cent c.i. 1.1–1.9)) and microscopic inflammation of both proximal and distal resection margins (HR 2.2 (95 per cent c.i. 1.2–3.9)) were associated with endoscopic and/or radiologic recurrence. Postoperative prophylaxis with monotherapy of immunomodulators (HR 0.6 (95 per cent c.i. 0.4 to 0.9)) or in combination with biologicals (HR 0.2 (95 per cent c.i. 0.05 to 0.4)) and other indication for ICR (HR 0.1 (95 per cent c.i. 0.01 to 0.93)) were associated with a lower risk of endoscopic and/or radiological recurrence. The estimated cumulative incidence of endoscopic and/or radiologic recurrence after ICR is displayed for a selection of covariates (smoking status, biological use prior to ICR, disease localization, microscopic inflammation of the resection margins, disease behaviour and postoperative prophylactic medication; all other covariates set to reference values) in *Fig. 2*.

## Discussion

In this multicentre study, patients with an indication for ICR shortly after CD diagnosis or who were diagnosed following ICR had the most favourable postoperative disease course, although differences were small. Studying prognosis after correction for possible confounding factors revealed a 4 per cent lower endoscopic recurrence rate, 8 per cent lower frequency of escalation of IBD medication and a 14 per cent lower long-term re-resection rate in patients who had ICR shortly after diagnosis (0–1 months), as compared to patients with ICR 1 year after diagnosis. The prognosis stabilized for patients who had undergone ICR approximately 4 months after diagnosis and remained stable throughout follow-up. Therefore, a benefit of early ICR after CD diagnosis to change the disease course is not substantiated by the data of this cohort study.

The group of patients with urgent ICR shortly after diagnosis comprises mainly patients who are diagnosed with severe stricturing disease or penetrating disease complications, and in a small number of patients the primary indication for ICR was the suspicion of appendicitis or diagnostic laparoscopy. Unfortunately, the number of patients in this study did not allow the study of subgroups within this mixed patient population. A beneficial prognosis for this group of patients may seem counterintuitive, because they have a delayed CD diagnosis when complications have already developed, which seems substantiated by a higher median age of 40 years at CD diagnosis. However, the finding of a beneficial disease course for this subgroup has also been shown in previous cohorts. An Italian retrospective study in 207 CD patients observed that surgery performed in an acute or subacute setting at diagnosis of CD was associated with a significantly lower incidence of clinical recurrence (defined as the need for corticosteroids in the presence of endoscopic or radiologic disease) during long-term follow-up, as compared to a postponed ICR^18^. Two other reports found lower overall surgery rates in patients with limited surgery immediately at diagnosis as compared to patients without surgery at diagnosis, although this result did not persist after propensity-score matching in one of the studies^19,24^. In addition, lower use of biologics in patients with ICR within 1 month after diagnosis has been reported^20^. With regard to the clinical implication of these findings, the identification of the subgroup of CD patients with an urgent ICR at diagnosis will not influence a decision in timing of ICR in clinical practice, because the decision to proceed to intestinal resection at first manifestation is often inevitable. Furthermore, it should be taken into account that differences in endoscopic recurrence rates are small and confidence intervals overlap. However, these findings may influence decisions on the need for prophylactic medication, in which the more favourable disease course of this subgroup may be taken into account.

Investigating the effect of timing of ICR on postoperative prognosis as a continuous variable in a multivariable model instead of predefined categories has allowed us to assess changes in recurrence rate throughout the CD disease course. Interestingly, recurrence rates increased strongly for patients with ICR more than 1 month after diagnosis and peak between 1 and 2 months after diagnosis for escalation of IBD medication and re-resection. One could speculate that this spike might be explained by a subset of CD patients with a high proportion of complicated disease (82.4 per cent stricturing or penetrating) but also with more extensive disease localization (47.1 per cent ileocolonic disease), in whom ICR was postponed at diagnosis but appeared inevitable shortly after, resulting in a poorer postoperative disease course characterized by symptoms necessitating the start or switch of IBD medication and a higher chance of re-resection. However, importantly, due to a small number of patients (*N* = 17) in this timeframe and wide confidence intervals, these results should be interpreted with caution and no further statistical analyses could be performed.

Postoperative prognosis stabilized for patients who had undergone ICR approximately 4 months after diagnosis and remained stable throughout follow-up. No evidence was found of an advantage for early surgery more than 1 month after diagnosis with regard to endoscopic and/or radiologic recurrence, escalation of IBD medication and re-resection. These study results indicate that timing of primary ICR in CD is not associated with postoperative prognosis. Although these findings do not provide guidance for the decision on when to perform ICR (early or late after CD diagnosis), they support the conception that early and late ICR may be considered equally effective alternatives with regard to long-term prognosis. Previous results from the LIR!C trial showed ICR to be comparable to step-up to infliximab in terms of quality of life and costs in biological-naïve patients, with a median disease duration of 13 months^14,15^. In a follow-up study, survival without additional treatment (escalation of IBD medication or resection) was comparable between both groups^25^. In addition, previous studies on timing of resection from the patients’ perspective showed patients to be satisfied with the results of the resection, and up to 74 per cent of patients would have preferred the resection to be performed earlier^26,27^. Naturally, this decision requires a multidisciplinary approach, taking into account disease extent and localization, disease behaviour, patient-related factors, including nutritional status and performance status, and patient preference.

Factors associated with postoperative recurrence after ileocolonic resections in CD have been studied extensively, mostly in retrospective cohorts and often with contradicting results. In contrast to most studies, this study only concerns patients after primary ileocecal resection. Active smoking status is an established individual risk factor for postoperative recurrence in CD with a two-fold risk of clinical recurrence and 2.5-fold risk of surgical recurrence^9^. Unexpectedly, smoking was not associated with an increased risk of endoscopic recurrence in our study. A possible explanation is the interaction of smoking with other risk factors in multivariable analysis. Next to active smoking, current guidelines consider penetrating disease behaviour as a risk factor^8,10^. However, conflicting data exist with regard to penetrating disease behaviour and the risk of early recurrence^8,12^. In this study, penetrating disease behaviour was not associated with endoscopic and/or radiologic recurrence. These differences might be explained by smaller population size and lower event rate in our study. Microscopic inflammation of both the proximal and distal margin was associated with an increased hazard of endoscopic recurrence. This is in line with recent reports showing that microscopic inflammation of the resection margins is associated with postoperative recurrence^28–31^. Furthermore, our study showed that patients receiving combination therapy with an immunomodulator and biological were at 20 per cent lower risk of endoscopic and/or radiologic recurrence after 18 months. This is consistent with published network meta-analyses, and may be a valuable option for patients with a high risk of recurrence^32,33^.

Although this study is based on a large data set of patients with a long-term follow-up, a few limitations of this study need to be taken into account, mostly inherent to its retrospective design. First, the analysis on the indication for ICR as a possible prognostic factor could benefit from further refinement of the subgroup of patients with refractory disease. For instance, the prognosis of CD irresponsive to a limited trial of one biologic may differ from CD failing multiple biologics. Unfortunately, subanalysis on the effect of timing of ICR in the population with prior biological use is hampered by the low number of patients exposed to a biological with ICR early after diagnosis. Second, postoperative follow-up was not standardized and therefore endoscopy was not routinely performed in all patients. Endoscopies may have been performed primarily in symptomatic patients, especially during earlier calendar years. As a result, endoscopic recurrence rates might be overestimated. Since a sensitivity analysis on the total study population results in similar observations on prognosis with regard to timing of ICR (*Supplementary material*), it can be speculated that the influence of this selection of patients on the primary analysis is limited. Thirdly, timing and indication of ICR were chosen by discretion of the treating physicians, which represents real-world clinical data, but also creates heterogeneity in the patient population. It cannot fully be excluded that the association between timing of ICR and postoperative prognosis was influenced by other factors that lead to the decision when to perform ICR which were not available in the data of the present study. However, the effect of this limitation was minimized by correcting for confounding factors like disease severity, age and indication for ICR in the present Cox model. Furthermore, although both secondary and tertiary centres were included, the largest proportion of patients (58 per cent) was treated in a tertiary or academic setting.

Overall, the short- and long-term postoperative prognoses are unaffected by the timing of ICR, when corrected for confounding factors. Therefore, the timing of ICR in CD is not associated with a change of the disease course and should not be weighed in clinical decisions.

## Supplementary Material

zrad097_Supplementary_Data

## Data Availability

The data underlying this article will be shared on reasonable request to the corresponding author.
