# Supplementary material for: Impact of timing of primary ileocecal resection on prognosis in patients with Crohn’s disease
Source: BJS Open. 2023 Sep 29;7(5):zrad097. doi: 10.1093/bjsopen/zrad097 (PMC10540509; doi:10.1093/bjsopen/zrad097)
Supplement: zrad097_Supplementary_Data [file zrad097_supplementary_data.docx]

**The impact of timing of primary ileocecal resection on prognosis in patients with Crohn's disease**

E.M.J. Beelen, MD.^1^ J.H.C. Arkenbosch, MD.^1^ N.S. Erler, PhD.^2^, J.A.M. Sleutjes, MD.^1^ F. Hoentjen, MD PhD.^3^ A.G.L. Bodelier, MD PhD.^4^ G. Dijkstra, MD PhD.^5^, M. Romberg-Camps, MD PhD.^6^ N.K. de Boer, MD PhD.^7^ L.P.S. Stassen, MD PhD.^8^ A.E. van der Meulen, MD PhD.^9^ R. West, MD PhD.^10^ O. van Ruler, MD PhD.^11^ C.J. van der Woude, MD PhD.^1^ A.C. de Vries, MD PhD.^1^

*On behalf of the Dutch Initiative on Crohn and Colitis (ICC)*

1. Department of Gastroenterology and Hepatology, Erasmus University Medical Center, Rotterdam, the Netherlands

2. Department of Biostatistics, Erasmus University Medical Center, Rotterdam, the Netherlands
3. Department of Gastroenterology and Hepatology, Radboud University Medical Center, Nijmegen, the Netherlands
4. Department of Gastroenterology and Hepatology, Amphia hospital, Breda, the Netherlands

5. Department of Gastroenterology and Hepatology, University Medical Center Groningen, Groningen, the Netherlands

6. Department of Gastroenterology and Hepatology, Zuyderland Medical Center, Sittard-Geleen, the Netherlands

7. Department of Gastroenterology and Hepatology, AGEM Research institute, Amsterdam University Medical Center, Vrije Universiteit Amsterdam, Amsterdam, the Netherlands

8. Department of Surgery, Maastricht University Medical Center, Maastricht, the Netherlands

9. Department of Gastroenterology and Hepatology, Leiden University Medical Center, Leiden, the Netherlands

10. Department of Gastroenterology and Hepatology, Fransiscus Gasthuis & Vlietland, Rotterdam, the Netherlands

11. Department of Surgery, IJsselland hospital, Capelle aan den IJssel, the Netherlands

**Corresponding author**

A.C. de Vries (MD, PhD)

Department of Gastroenterology and Hepatology

Erasmus University Medical Center

PO Box 2040, 3000 CA

Rotterdam, The Netherlands

Email: a.c.devries@erasmusmc.nl

Telephone: 0031- 107 030 792

**Supplementary Materials - Index**

| **Supplementary Figures and Tables** |  |
| --- | --- |
| Figure S1  Figure S2 | *page 2*  *page 3* |
| Table S1  Figure S3  Figure S4 | *page 4*  *page 6*  *page 7* |
|  |  |
|  |  |

**Supplementary Figures and Tables**

**Figure S1**

Patient inclusion over time


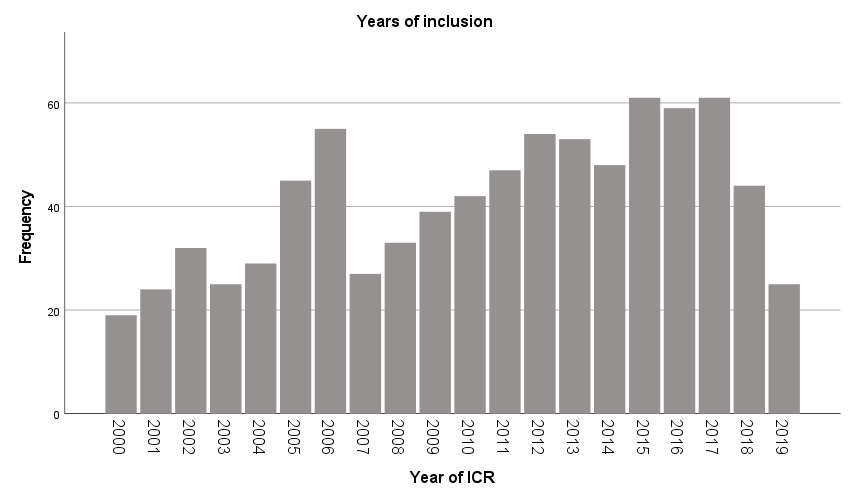


**Figure S2** Vizualisation of the effect of the timing of ileocecal resection (ICR) on recurrence, modeled with natural cubic splines. Shown is the expected cumulative incidence 18 months after ICR for endoscopic and/or radiologic recurrence (n=527) and escalation of IBD medication (n=822) (panels A1 and B1), and after total-follow up (17 years) for re-resection (n=822) (panel C1), unadjusted for other covariates. The shaded area represents the corresponding 95% CI. The inset plots (A2-C2) zoom in on the first 12 months.


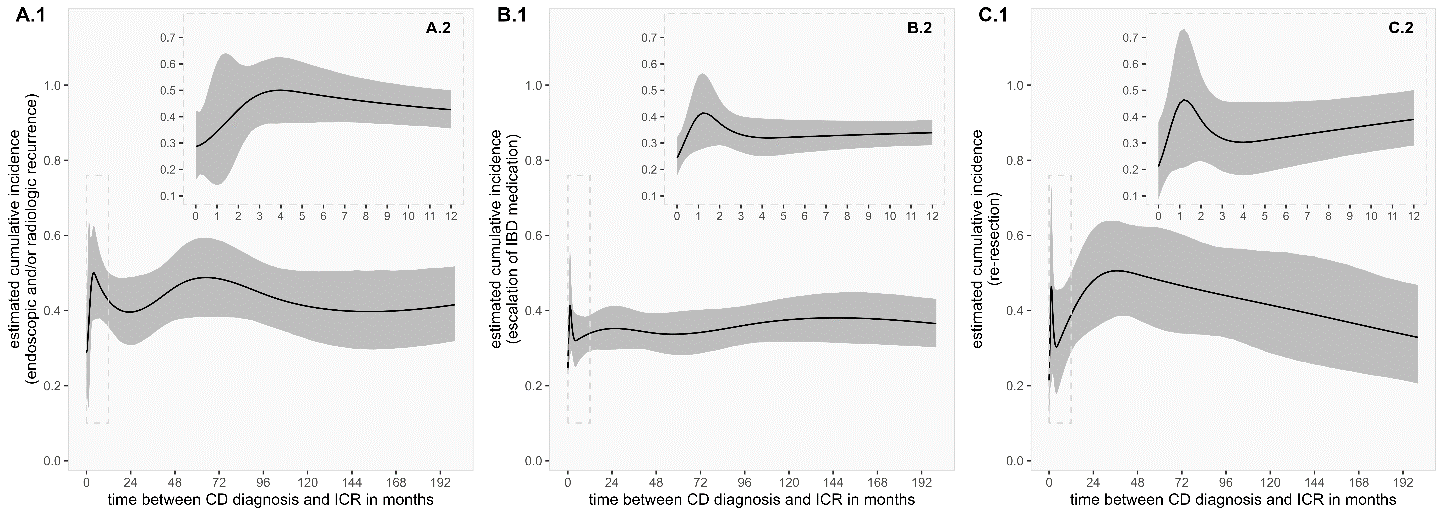


**Table S3.** Characteristics of the study population subdivided according to timing of ICR: ICR within 0-1 months after CD diagnosis, ICR within 4 months after diagnosis and ICR more than 4 months after diagnosis. Shown are median (interquartile range) or count (proportion).

|  | | ICR after 0-1 months (N=78) | ICR after 1-4 months (N=52) | ICR after >4 months (N=692) | P-value |
| --- | --- | --- | --- | --- | --- |
| Gender, male | | 38 (48.7%) | 26 (50.0%) | 253 (36.6%) | 0.024 |
| Age at ICR in years, median | | 40.4 (24.4 – 53.7) | 30.1 (22.9 -44.0) | 32.0 (24.1 – 43.8) | 0.080 |
| Positive family history of IBD | | 14 (18.0%) | 10 (19.2%) | 143 (20.7%) | 0.881 |
| *missing* | | *28 (35.9%)* | *17 (32.7%)* | *230 (33.2%)* |  |
| Active smoking | | 27 (34.6%) | 14 (26.9%) | 244 (35.3%) | 0.567 |
| *missing* | | *6 (7.7%)* | *5 (9.6%)* | *42 (6.1%)* |  |
| Body Mass Index, median | | 20.5 (18.1 – 23.4) | 20.8 (18.7 -24.2) | 22.9 (20.0 – 26.0) | <0.001 |
| *missing* | | *27 (34.6%)* | *19 (36.5%)* | *193 (27.9%)* |  |
| Age at diagnosis in years | | 40.4 (24.4 – 53.7) | 29.9 (22.8 – 43.7) | 24.7 (19.4 – 35.2) | <0.001 |
| Montreal L | Ileum | 56 (71.8%) | 31 (59.6%) | 436 (63.0%) | 0.256 |
|  | Ileocolonic | 22 (28.2%) | 21 (40.4%) | 256 (37.0%) |  |
| Montreal B | Luminal | 12 (15.4%) | 7 (13.5%) | 158 (22.8%) | <0.001 |
|  | Stricturing | 30 (38.5%) | 22 (42.3%) | 348 (50.3%) |  |
|  | Penetrating | 36 (46.2%) | 23 (44.2%) | 186 (26.9%) |  |
| Peri-anal fistulas | | 2 (2.6%) | 2 (3.8%) | 93 (13.4%) | 0.003 |
| Biological use prior to ICR | | 1 (1.3%) | 5 (9.6%) | 360 (52.0%) | <0.001 |
| Immunomodulator use prior to ICR | | 1 (1.3%) | 11 (21.2%) | 529 (76.4%) | <0.001 |
| Indication for ICR | Non-complicated disease refractory to therapy / instead of step-up | 2 (2.6%) | 8 (15.4%) | 214 (30.9%) | <0.001 |
|  | Stricture | 36 (46.2%) | 23 (44.2%) | 339 (49.0%) |  |
|  | Fistula/abscess | 27 (34.6%) | 21 (40.4%) | 137 (19.8%) |  |
|  | Other | 13 (16.7%) | 0 (0%) | 2 (0.3%) |  |
| Surgical approach | Laparotomy | 53 (68.0%) | 32 (61.5%) | 277 (40.0%) | <0.001 |
|  | Laparoscopy | 20 (25.6%) | 19 (36.5%) | 387 (55.9%) |  |
|  | *missing* | *5 (6.4%)* | *1 (1.9%)* | *28 (4.1%)* |  |
| Microscopic inflammation of resection margin | No inflammation | 35 (44.9%) | 31 (59.6%) | 320 (46.2%) | 0.199 |
|  | Proximal inflammation | 15 (19.2%) | 4 (7.7%) | 116 (16.8%) |  |
|  | Distal inflammation | 3 (3.8%) | 2 (3.9%) | 32 (4.6%) |  |
|  | Both sides inflammation | 1 (1.3%) | 1 (1.9%) | 39 (5.7%) |  |
|  | *missing* | *24 (30.8%)* | *14 (26.9%)* | *185 (26.7%)* |  |
| Length of resected segment in cm, median | | 28.0 (18.0 – 40.0) | 24.0 (20.1 – 29.3) | 24.5 (18.0 -33.0) | 0.211 |
| *missing* | | *9 (11.5%)* | *4 (7.6%)* | *50 (7.2%)* |  |
| Type anastomosis | Side-to-side | 51 (65.4%) | 31 (59.6%) | 515 (74.4%) | 0.602 |
|  | End-to-side | 4 (5.1%) | 3 (5.8%) | 44 (6.4%) |  |
|  | End-to-end | 9 (11.6%) | 9 (17.3%) | 56 (8.1%) |  |
|  | *missing* | *14 (17.9%)* | *9 (17.3%)* | *77 (11.1%)* |  |
| Prophylactic postoperative medication | None/5ASA/corticosteroid | 68 (87.2%) | 40 (76.9%) | 406 (58.6%) | <0.001 |
|  | Immunomodulator | 7 (9.0%) | 10 (19.2%) | 170 (24.6%) |  |
|  | Biological | 1 (1.3%) | 1 (1.9%) | 67 (9.7%) |  |
|  | Immunomodulator and biological | 2 (2.6%) | 1 (1.9%) | 47 (6.8%) |  |
|  | *missing* | *0 (0%)* | *0 (0%)* | *2 (0.3%)* |  |

**Figure S3.** Vizualisation of the effect of timing of ileocecal resection (ICR) on endoscopic and/or radiologic recurrence, where endoscopic recurrence was defined as Rutgeerts’ score ≥i3. Shown is the expected cumulative incidence of endoscopic and/or radiologic recurrence at 18 months after ICR for cut-off Rutgeerts’ score ≥i2b (panel A) and ≥i3 (panel B).


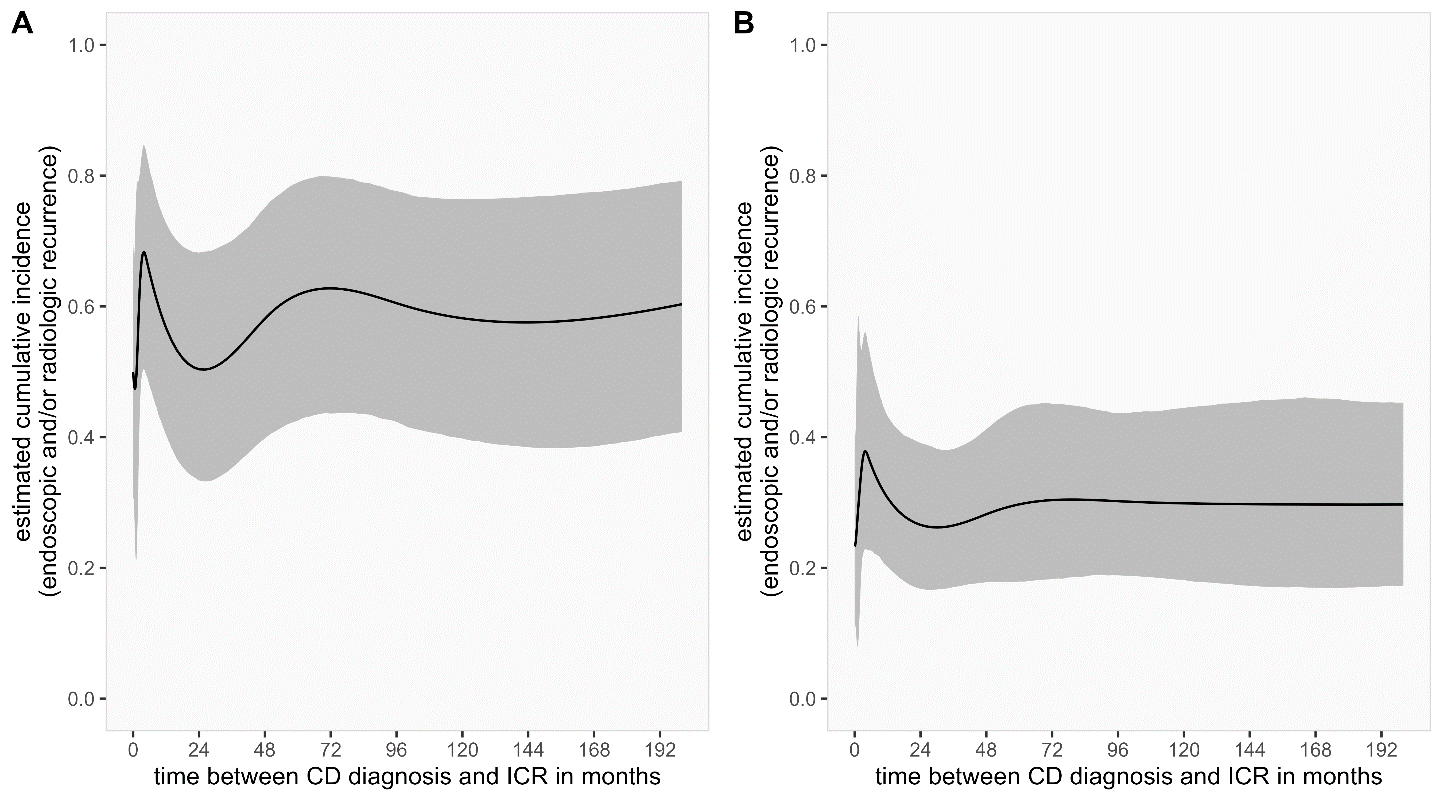


**Figure S4.** Vizualisation of the effect of the timing of ileocecal resection (ICR) on recurrence, modelled with natural cubic splines. Shown is the expected cumulative incidence 18 months after ICR for endoscopic and/or radiologic recurrence, for the total study population (n=822). The inset plot zooms in on the first 12 months

**
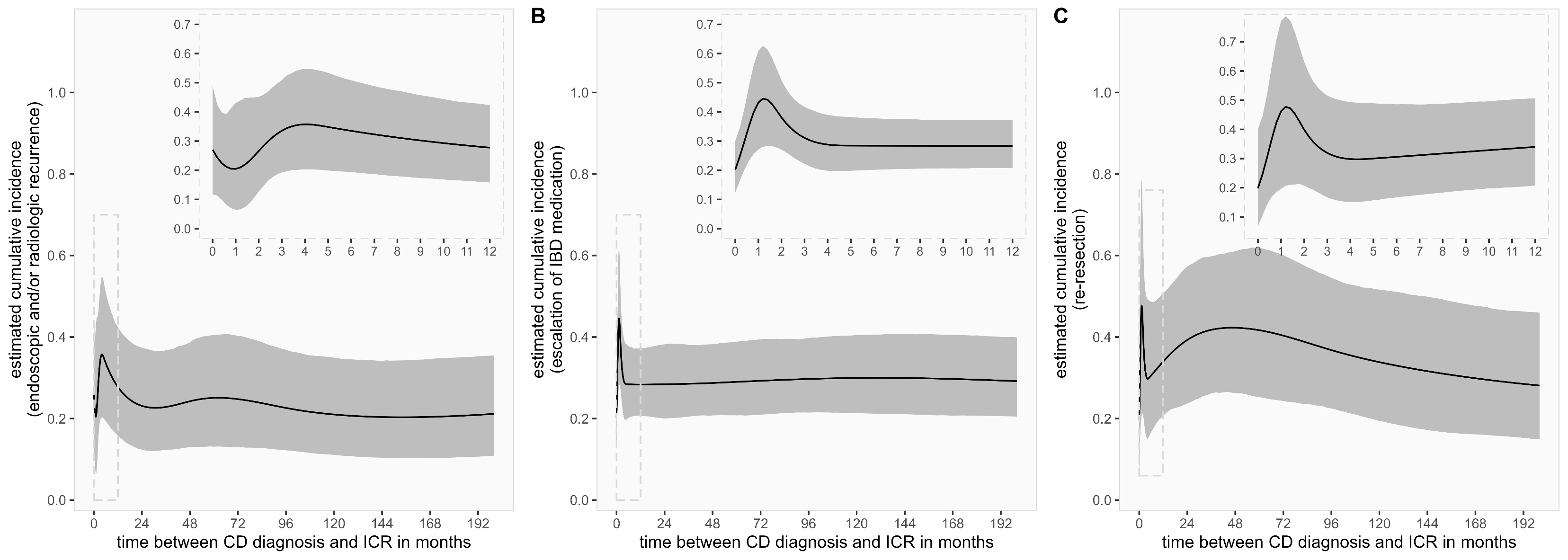
**
